# Del Nido Cardioplegia in Adult CABG: Clinical and Electrophysiological Advantages

**DOI:** 10.21470/1678-9741-2025-0269

**Published:** 2026-09-10

**Authors:** Vasileios Leivaditis, Nikolaos G. Baikoussis

**Affiliations:** 2 Department of Cardiac Surgery, Ippokrateio General Hospital of Athens, Athens, Greece

We read with great interest the article by Toz et al. comparing del Nido cardioplegia (DNC) and conventional blood cardioplegia (BC) in patients undergoing isolated coronary artery bypass grafting (CABG), with a focus on the incidence of postoperative atrial fibrillation (POAF). The findings of their study are not only relevant but also timely, as POAF remains one of the most frequent complications following cardiac surgery, affecting up to 50% of patients depending on the population and monitoring methods used^[^1^]^.

Their central conclusion - that DNC is associated with significantly lower POAF rates on postoperative days one, five, and 30 compared to BC - is clinically meaningful and supported by a clear methodological approach. The authors have made a valuable contribution to an area where evidence remains inconsistent and somewhat fragmented.

The reduction in POAF observed with DNC is likely multifactorial. The composition of the DNC solution is specifically designed to provide both myocardial protection and electrical stability.

The beneficial effects of DNC are attributed to the synergistic action of its main additives: lidocaine provides electrical stabilization, magnesium counteracts calcium overload, and mannitol reduces edema and oxidative stress. Together, these components enhance myocardial protection and rhythm stability, offering a plausible explanation for the lower incidence of POAF^[^2^,^3^]^.

In the study by Toz et al.^[^1^]^, patients receiving DNC experienced shorter aortic cross-clamping and cardiopulmonary bypass times, along with markedly lower defibrillation rates (12.9% *vs.* 74.3%, *P* < 0.001). This reflects not only better myocardial preservation but also a smoother intraoperative course. Fewer interruptions for redosing cardioplegia may result in more efficient surgery, reduced ischemic intervals, and less myocardial manipulation.

Interestingly, the benefit of DNC extended beyond the operating room. Patients in the DNC group had shorter intubation times and reduced length of stay in the intensive care unit. While these were not the primary endpoints of the study, they hint at broader benefits in terms of recovery and resource utilization. This is consistent with prior reports. Guajardo Salinas et al., for example, found that DNC reduced the volume of cardioplegia required and the need for defibrillation after cross-clamp removal in a cohort of adults undergoing primary CABG^[^4^]^.

The retrospective design of the study naturally limits its ability to infer causation, but the authors minimized confounding through balanced inclusion criteria and detailed intraoperative protocols. All patients had a left ventricular ejection fraction > 30% and were in sinus rhythm preoperatively. They excluded those undergoing emergent procedures or with a history of atrial fibrillation, thus aiming to isolate the cardioplegia effect.

One methodological aspect we feel deserves clarification is the detection of POAF on postoperative day 30. The authors mention routine follow-ups and electrocardiograms (ECGs), but do not describe whether continuous or ambulatory monitoring was employed. Given that POAF is frequently paroxysmal and asymptomatic - especially in the subacute period - there is a risk of underdiagnosis without prolonged monitoring. Studies such as that by Gillinov et al.^[^5^]^ have shown that intermittent ECGs may miss a significant proportion of atrial arrhythmias unless continuous telemetry or Holter monitoring is used.

Moreover, while baseline characteristics were comparable, we noted that variables known to influence POAF - such as left atrial size, preoperative use of beta-blockers, or anti-inflammatory agents - were not reported. Future studies would benefit from including such data, especially if prospective in design.

While the results are highly encouraging, their generalizability may be somewhat limited by the specific patient population included in the study. The exclusion of higher-risk patients, such as those undergoing reoperations or with severe ventricular dysfunction, makes it difficult to extend these findings to more complex surgical cohorts. It would be useful to evaluate whether DNC maintains its advantages in patients of advanced age, with frailty, or with prior myocardial infarction.

Nonetheless, this study adds weight to the growing argument for broader use of DNC in adult cardiac surgery. Schutz et al.^[^6^]^, in a propensity-matched study of over 800 patients, similarly found that DNC was associated with a lower incidence of POAF and ventricular arrhythmias compared to BC. In both that study and the one by Toz et al.^[^1^]^, these benefits were achieved without increasing operative mortality or other adverse events.

There are also practical benefits to consider. The single-dose administration of DNC reduces the number of surgical pauses required to re-administer cardioplegia, which can help optimize operating room time and reduce the potential for technical errors. Especially in high-volume centers or during complex procedures, this operational simplicity is not trivial.

In conclusion, the study by Toz et al.^[^1^]^ provides compelling evidence that DNC is not only a safe and effective myocardial protection strategy in adult CABG surgery but may also confer measurable benefits in terms of rhythm stability and recovery. The significantly lower incidence of POAF, shorter operative times, and decreased need for defibrillation all suggest that DNC offers a promising alternative to traditional BC.

Future prospective, multicenter studies with standardized postoperative rhythm surveillance and broader patient inclusion criteria will be important to confirm and expand upon these findings. Until then, this study adds a strong voice to the case for adopting DNC more widely in adult surgical practice.

## Artificial Intelligence Usage

The authors declare that no artificial intelligence tool was used in the preparation of this article.
